# Spatiotemporal and Demographic Patterns of West Nile Neuroinvasive Disease in Vojvodina, Serbia, 2012–2025

**DOI:** 10.3390/v18030312

**Published:** 2026-03-02

**Authors:** Snežana Medić, Tatjana Pustahija, Aleksandra Patić, Siniša Sević, Mioljub Ristić, Gordana Kovačević, Athanasios Tsakris, Cleo Anastassopoulou, Zagorka Lozanov-Crvenković

**Affiliations:** 1 Faculty of Medicine, University of Novi Sad, Hajduk Veljkova 3, 21000 Novi Sad, Serbia; tatjana.pustahija@mf.uns.ac.rs (T.P.); aleksandra.patic@mf.uns.ac.rs (A.P.); sinisa.sevic@mf.uns.ac.rs (S.S.); mioljub.ristic@mf.uns.ac.rs (M.R.); gordana.kovacevic@izjzv.org.rs (G.K.); 2 Institute of Public Health of Vojvodina, Futoška 121, 21000 Novi Sad, Serbia; 3 Infectious Diseases Clinic, University Clinical Center of Vojvodina, Hajduk Veljkova 1, 21000 Novi Sad, Serbia; 4 Department of Microbiology, Medical School, National and Kapodistrian University of Athens, 115 27 Athens, Greece; atsakris@med.uoa.gr (A.T.); cleoa@med.uoa.gr (C.A.); 5 Department of Mathematics and Informatics, Faculty of Science, University of Novi Sad, Trg Dositeja Obradovića 3, 21000 Novi Sad, Serbia

**Keywords:** West Nile neuroinvasive disease (WNND), West Nile virus (WNV), public health, vector-borne infections, zoonoses, surveillance, diagnostics

## Abstract

West Nile neuroinvasive disease (WNND) causes substantial morbidity in endemic regions, yet data on its burden in Serbia remain limited. We conducted a retrospective, population-based study of WNND cases reported in Vojvodina Province, Serbia, from 2012 to 2025. Incidence and mortality trends were analysed by year, residence, age, sex, and week of symptom onset. Multivariable logistic regression was used to identify predictors of fatal outcome. Of 1337 suspected cases, 557 (41.66%) met the WNND case definition (530 confirmed, 27 probable cases) and 98.9% were autochthonous. The mean annual incidence was 2.17/100,000 (95% CI 0.60–3.75), ranging from 0.48/100,000 (2015) to 10.31/100,000 (2018), with additional peaks in 2013 and 2022. Cases clustered predominantly in epidemiological weeks 31–34. The mean mortality was 0.28/100,000 (95% CI 0.02–0.53) and the mean case fatality rate was 12.93% (95% CI 10.14–15.71%). Incidence increased with age, peaking at 5.97/100,000 in those 70–79 years; highest mortality occurred in ≥80 years (1.78/100,000). All districts reported cases, with the highest incidence and mortality in South Banat. Higher Charlson Comorbidity Index, cardiovascular disease, diabetes and malignancy independently predicted fatal outcome. WNND remains a significant public health problem in Vojvodina, requiring improved surveillance, targeted prevention, and early treatment of high-risk patients.

## 1. Introduction

West Nile fever (WNF), a vector-borne infectious disease, has become endemic in Europe and is gradually expanding its geographic range into previously unaffected, non-endemic areas [1,2]. The natural transmission cycle of West Nile virus (WNV) occurs primarily among *Culex* spp. mosquitoes and wild birds, while humans are considered accidental dead-end hosts [3,4]. WNV circulates seasonally across Europe with outbreaks concentrated in the Mediterranean and Southeast areas, where genetic heterogeneity, notably co-circulation and local evolution of lineages 1 and 2 shapes outbreak patterns [5,6,7]. Enzootic bird–mosquito cycles sustain transmission with spillover to horses and humans while repeated detections in wild birds, mosquitoes, and ungulates confirm endemic circulation in Southeast Europe, including Serbia [5,6,7]. Human WNV infection is predominantly asymptomatic; however, among symptomatic individuals, clinical manifestations range from a self-limiting febrile illness (approximately 20% of cases) to West Nile neuroinvasive disease (WNND) in fewer than 1% of infected individuals [8]. WNND presents as meningitis, encephalitis, or acute flaccid paralysis, predominantly in older or immunocompromised adults [6,7].

Outbreaks of WNF are frequently accompanied by a substantial burden of WNND in the human population, which carries an overall case-fatality rate (CFR) of approximately 10%, rising up to 20% or more among elderly individuals and those with compromised immune systems [9,10,11]. Rare secondary routes of WNV transmission include transfusion of blood products, organ transplantation, transplacental transmission, breastfeeding, and accidental exposure among laboratory personnel [12,13,14].

Potential drivers of WNF emergence in humans include climate change, which has expanded ecologically suitable areas for WNV circulation and increased the density of infected mosquitoes. Additional contributing factors include specific local environmental conditions and rising human population density [15]. During the 1990s and the first two decades of the 21st century, climatic conditions were particularly favorable for the spread of WNV in Central and Southeastern Europe [16,17]. Following the introduction of enhanced surveillance for WNF in 2012, mainly focused on WNND cases, the Republic of Serbia has consistently ranked among the countries with the highest reported human WNF incidence in the region [18,19,20,21].

The determinants of WNF occurrence vary substantially across endemic regions, reflecting complex interactions among ecological, climatic, vector-related, and sociodemographic factors that influence WNV transmission [15]. Accordingly, a thorough understanding of region-specific determinants is essential for the development of effective public health strategies aiming to reduce the risk of WNF outbreaks. We present a comprehensive epidemiological analysis of human WNND cases in the Autonomous Province of Vojvodina, Northern Serbia—based on national surveillance data for a region representing 26.2% of Serbia’s population [22]. The study aims to characterize long-term demographic, temporal, seasonal, and spatial patterns of WNND in the region from 2012 to 2025 and to identify predictors of fatal outcomes, thereby providing evidence to support improved risk assessment and prevention strategies.

## 2. Materials and Methods

### 2.1. Surveillance of WNF in Human Population

In Serbia, notification of WNF cases was mandatory from 2012 to 2016, in accordance with the International Health Regulations [23] and has since been regulated by national legislation [24]. Active surveillance is conducted during the WNV transmission season (1 June–15 November), while passive surveillance is performed during the remainder of the year. Surveillance is hospital-based and implemented in collaboration with district Institutes of Public Health (IPHs), the Institute of Public Health of Vojvodina (IPHV) at the provincial level, and the Institute of Public Health of Serbia at the national level. The national case definition of WNF in Serbia [25] is aligned with the European Union (EU) case definition [26] and formally encompasses all clinical forms of WNV infection, including non-neuroinvasive disease and WNND. However, throughout the entire study period (2012–2025), surveillance activities in Vojvodina were exclusively focused on WNND. Although WNF and WNND are virologically indistinguishable and should be considered equally when specific diagnostic methods are available, the surveillance strategy was oriented toward neuroinvasive disease because it represents a more specific indicator of clinically apparent WNV activity in the human population. Accordingly, WNND cases were classified as probable or confirmed based on the national case definition, restricted to cases presenting with neurological manifestations. Clinical criteria included fever and/or neurological symptoms consistent with encephalitis or meningitis. Acute flaccid paralysis (AFP) was not specifically included in the national WNND definition during the study period. Laboratory confirmation required at least one of the following: isolation of WNV from blood or cerebrospinal fluid (CSF); detection of WNV nucleic acid in blood or CSF; detection of WNV-specific IgM antibodies in CSF; or high titers of WNV-specific IgM antibodies with concurrent detection of WNV-specific IgG antibodies and confirmation by neutralization test. Detection of WNV-specific antibodies in serum alone was considered laboratory evidence for a probable case. Probable cases were defined as individuals fulfilling the clinical criteria together with epidemiological linkage and/or laboratory evidence for a probable case, while confirmed cases met the laboratory criteria for case confirmation. All WNND cases were reported by clinicians to the IPHs and entered into the surveillance database, followed by case investigations conducted by epidemiologists using a standardized questionnaire. The IPHs coordinated the public health response during the WNF transmission season.

### 2.2. Study Setting and Data Collection

A retrospective, population-based study was conducted using surveillance data on probable and confirmed human WNND cases recorded in Vojvodina, Serbia, between 2012 and 2025, including imported cases. Cases residing outside Vojvodina or not meeting the national case definition criteria for probable or confirmed WNND cases were excluded.

The study area comprised the Vojvodina province (21,614 km^2^; population 1,740,230 in 2022) [22], which is administratively divided into seven districts and 45 municipalities. During the study period, the population declined by 9.3%, reflecting national demographic trends of negative natural change and net emigration together with an aging population (proportion of individuals aged ≥65 years increased from 16.5% in 2011 to 21.7% in 2022) [27].

Data on WNND cases in Vojvodina were obtained from the IPHV surveillance databases and analysed. The data set included demographic characteristics (age, sex, district and municipality of residence), date of symptom onset, clinical presentation of WNND, type of positive laboratory diagnostic test and disease outcome (recovered or death) at the time of hospital discharge. In addition, both the number and type of comorbidities were recorded, including cardiovascular diseases (excluding myocardial infarction), hypertension, diabetes mellitus, malignancy (excluding leukemia), chronic obstructive pulmonary disease, chronic kidney disease, chronic rheumatic disease, and cerebrovascular insult. Myocardial infarction and leukemia were recorded as separate comorbidities. Furthermore, data on the time and location of recent mosquito bites, travel history and blood donation within 14 days before symptom onset, as well as blood transfusion or organ transplantation within 30 days before disease onset, were recorded. Information on prior vaccination against *Ortoflavivirus* infections were also documented.

### 2.3. Laboratory Methods

Upon clinical suspicion of WNND, patients’ samples (serum and/or CSF) were transported under cold chain conditions to the laboratory of the Department of Virology, IPHV, Novi Sad. For serological analysis, specimens were tested for the presence of WNV-specific IgM and IgG antibodies using commercial semi-quantitative Anti-WNV ELISA IgM and IgG kits (Euroimmun, Lübeck, Germany), according to the manufacturer’s instructions. Samples with IgM and IgG indeks values <0.8 were considered negative, values ≥0.8 and <1.1 were considered equivocal, and values ≥1.1 were considered positive.

For molecular analysis, RNA was automatically extracted from serum and CSF samples using the QIAamp Viral RNA Mini kit (Qiagen, Hilden, Germany) on the QIAcube platform or the SaMag™ Viral Nucleic Acid Extraction Kit (Sacace, Biotechnologies, Como, Italy) on the SaMag—12 System. Qualitative detection of WNV RNA was performed using either the WNV Real-TM kit (Sacace, Italy) or the Bosphore^®^ WNV Quantification Kit v2 (Anatolia Geneworks, Istanbul, Turkey). Amplification was carried out on the Applied Biosystems 7500 Real-Time PCR System or the QuantStudio 5 Real-Time PCR System (Thermo Fisher Scientific, Waltham, MA, USA). Confirmatory testing of IgM/IgG and/or PCR-positive WNV residual samples was performed at the National Reference Laboratory, Institute “Torlak”, Belgrade, as previously described [28]. Viral RNA confirmatory detection by real-time RT-PCR followed established WNV diagnostic protocols for clinical specimens in accordance with national standardized reference laboratory practices for WNV infection [29].

### 2.4. Data Analysis and Statistics

The data were summarized using descriptive statistics and registered human WNND cases were analysed chronologically, demographically, and spatially. Continuous variables were reported as means with 95% confidence intervals (95% CI) or as median with interquartile ranges (IQR; Q1–Q3), depending on the data distribution. Categorical variables were presented as counts and percentages (*n*, %).

Crude incidence rates were calculated as the number of newly diagnosed WNND cases per 100,000 inhabitants for each study year, using the mid-year population of Vojvodina as the denominator based on official statistical estimates [22]. Mean annual incidence and mortality rates of WNND were calculated, with 95% CIs estimated for both measures. Sex- and age-specific annual and mean incidence and mortality rates were computed for each WNF season using the following age groups: 0–9, 10–19, 20–29, 30–39, 40–49, 50–59, 60–69, 70–79, and ≥80 years.

For spatial analysis, maps of Vojvodina were generated to show annual WNND incidence and mean district-level incidence over the 14-year study period; 2020 was excluded because no cases were reported. Seasonal distribution of WNND cases was assessed using cumulative case counts by epidemiological (epi) week [30]. For seasonal stratification, the date of symptom onset was used to assign cases to epidemiological weeks, serving as an imperfect but standard proxy for the date of infection in human arboviral surveillance [31]. Weekly WNND cases were aggregated and converted into cumulative counts for each epi week and season. Analyses were restricted to the transmission season, defined as epi weeks 24–40, and further stratified into early (weeks 24–27), peak (weeks 28–35), and late (weeks 36–40) transmission periods, reflecting the typical temporal pattern of WNV activity in the region. Box-and-whisker plots were used to summarize the distribution of cumulative WNND case counts across seasons for each epi week, displaying the median, interquartile range (Q1–Q3), and overall variability.

The mortality rates per 100,000 inhabitants and the corresponding CFRs—defined as the proportion of WNND-related deaths among all reported WNND cases and expressed as percentages were calculated for each WNF season during the study period. Temporal trends in WNND incidence and mortality rates were assessed using joinpoint regression analysis by estimating the annual percent change (APC) for each identified segment and the average annual percent change (AAPC), along with corresponding 95% CIs. Trends were classified as statistically significant if the APC or AAPC differed from zero at the 0.05 significance level.

Mean annual proportions of WNND clinical forms (meningitis, meningoencephalitis, encephalitis, and unspecified WNND) were calculated as arithmetic means of yearly proportions over the study period, with corresponding 95% CIs. Medians with interquartile ranges (Q1–Q3) were also reported. Distribution normality was assessed using the Kolmogorov–Smirnov test. Associations between WNND clinical forms and mortality were assessed using Spearman’s rank correlation coefficient (ρ) to examine the relationship between annual numbers of cases for each clinical form and the annual CFR. Analyses were performed separately for each clinical form, with correlation strength interpreted using standard thresholds: |ρ| < 0.3 (weak), 0.3–0.6 (moderate), and >0.6 (strong).

Patients with WNND were classified according to outcome as survivors or non-survivors. Demographic characteristics and clinical variables, including age, sex, number of comorbidities, Charlson Comorbidity Index (CCI) [32,33] to quantify overall comorbidity burden, and individual comorbid conditions—were compared between the two groups. To identify factors associated with fatal outcomes, univariate logistic regression analysis was performed for each variable, and odds ratios (ORs) with 95% CIs were calculated. Variables showing statistical significance in univariate analysis were subsequently included in a multivariable logistic regression model to determine independent predictors of mortality. Results of the multivariable analysis were reported as adjusted ORs with corresponding 95% CIs. Statistical significance was defined as a *p*-value <0.05.

Statistical analyses were performed using Stata version 18 (StataCorp LLC, College Station, TX, USA) and Joinpoint regression software version 5.4.0. [34]. Model selection in joinpoint regression was based on the Monte Carlo permutation test, allowing a maximum of three joinpoints to identify significant changes in trend direction. IBM SPSS version 26.0 (IBM Corp., Armonk, NY, USA) was used for additional statistical analyses. Quantum GIS version 3.42.1 (QGIS Development Team) was employed for map generation.

### 2.5. Ethical Statement

The study was conducted within the framework of the national public health surveillance system. Collection of clinical samples for laboratory diagnosis was part of the routine diagnostic protocol and required only verbal informed consent from patients. Only aggregated, anonymized data were analysed and presented, with no patient-identifying information included. The study protocol was approved by the Ethics Committee of the IPHV on 26 December 2025 (Approval No. 01-2309/1-1).

## 3. Results

A total of 1337 suspected cases of WNND in Vojvodina were investigated between 2012 and 2025. Of these, 557 cases (41.66%) met the case definition criteria, including 530 laboratory-confirmed cases (95.15%) and 27 probable cases (4.85%) (Table 1). The vast majority of cases were classified as autochthonous (551/557; 98.92%)**.** All confirmed WNND cases tested positive for WNV–specific IgM antibodies in both serum and CSF. When expressed relative to the number of subjects tested for each assay, WNV-specific IgM was detected in 39.64% of paired serum and CSF samples. Among confirmed cases, 229/530 (43.21%) also had detectable WNV-specific IgG antibodies in serum (17.12% of those tested). Additionally, molecular testing among confirmed cases detected WNV RNA in 14.69% of tested CSF and in 19.80% of tested serum samples. PCR positivity rates in CSF and serum fluctuated over the study period, reaching their highest levels in 2018 (24.56% and 35.02%, respectively) and declining thereafter. All 27 probable cases showed WNV-specific IgM and IgG antibodies in serum (2.02% of those tested), but none demonstrated serological or molecular evidence of WNV infection in CSF.

Over the 14-year study period, the mean annual incidence of WNND was 2.17 per 100,000 population (95% CI: 0.60–3.75) (Table 2). WNND cases were recorded across all age groups except children aged 0–9 years. Patient ages ranged from 12 to 90 years, and the majority of cases occurred in individuals aged >60 years (n = 356; 63.91%). The mean age of WNND patients was 62.0 ± 15.0 years, with a median age of 65 years (IQR: 55–72). Male patients accounted for 63.02% of cases (Table 2), corresponding to a male-to-female incidence rate ratio of 1.79. Incidence increased progressively with age, peaking among individuals aged 70–79 years (5.97/100,000 population; 95% CI: 1.50–10.45). Fatal outcomes were observed exclusively in patients aged ≥40 years, with the highest age-specific mortality rate among those aged ≥80 years (1.78 per 100,000 population; 95% CI: 0.00–3.80).

WNND cases were reported in all districts of the province, with the highest mean incidence rate observed in South Banat (4.97 per 100,000; 95% CI: 1.16–8.77) and the lowest in North Bačka (0.57 per 100,000; 95% CI: 0.21–0.93) (Table 2).

**Figure 1:**
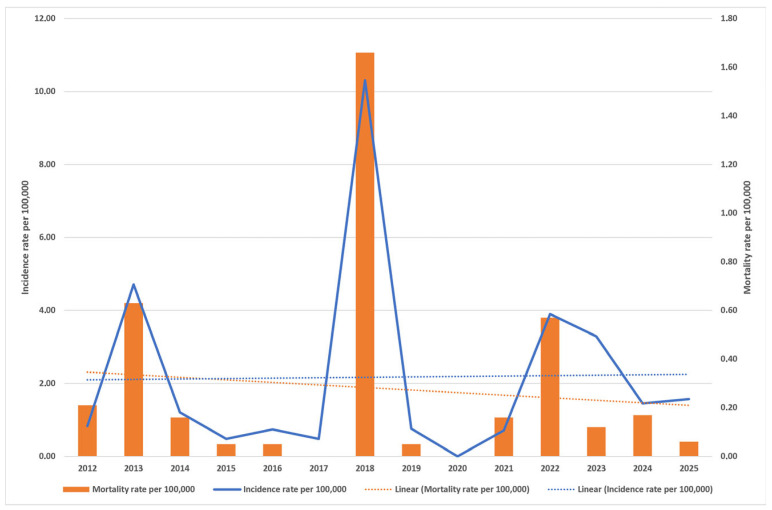
Annual incidence and mortality rates of WNND in Vojvodina, Serbia, 2012–2025.

WNND incidence varied considerably over time, ranging from a low of 0.48 per 100,000 population in 2015 to a peak of 10.31 per 100,000 population in 2018, with no cases reported in 2020 (Figure 1). Both incidence and mortality rates exhibited marked temporal fluctuations, with pronounced peaks observed in 2013, 2018, and 2022. A total of 72 WNND-related deaths were recorded during the study period, corresponding to a mean mortality rate of 0.28/100,000 population (95% CI 0.02–0.53) and a mean CFR of 12.93% (95% CI 10.14–15.71). Over the study period, the annual CFR showed an overall declining trend, decreasing from 25% in 2012 to 3.7% in 2025, with intermittent peaks observed in 2018 (16.15%) and 2021 (23.07%) and years with no recorded fatalities (2017 and 2020). The highest mortality rate was observed in 2018, reaching 1.66/100,000 population (Figure 1).

During the study period, the incidence rate of WNND demonstrated a moderate upward trend, while the mortality rate showed a slight downward trend. Joinpoint regression analysis supported an overall increasing trend in the incidence rate (AAPC_2012–2025_= 3.89%) and a decreasing trend in mortality (AAPC_2012–2025_= −12.10%) (Table S1). The analysis identified two incidence-rate trend segments, with a change in direction in 2020 from a decreasing trend (APC _2012–2020_= −28.72%) to an increasing trend (APC_2020–2025_= 89.82%) (Figure S1A). The mortality rate analysis revealed two joinpoints, resulting in three distinct trend segments (APC_2012–2020_ = −31.34%; APC_2020–2023_ = 218.89%; APC_2023–2025_ = −65.83%) (Figure S1B). None of the observed changes in incidence or mortality trends reached statistical significance (*p* > 0.05).

**Figure 2:**
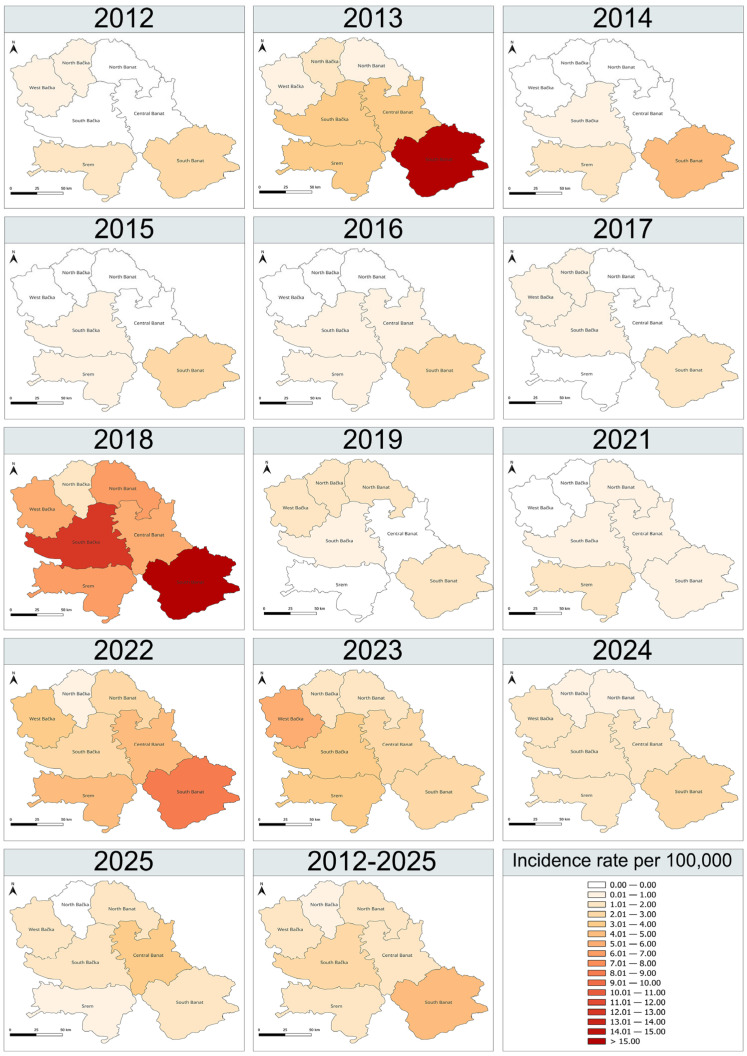
District-level annual incidence rates of WNND and the mean district-level incidence in the study region (Vojvodina Province, Serbia) for 2012–2025. Year 2020 is excluded because no cases were reported. Incidence is expressed per 100,000 population.

Among districts reporting WNND-related deaths, South Banat had the highest mean mortality rate, 6.77-fold higher than the lowest, observed in West Bačka. The spatial distribution of WNND is illustrated in maps showing annual district-level incidence rates per 100.000 population as well as average incidence for the 14-year study period, excluding 2020 (Figure 2). From 2012 to 2025, WNND cases were reported annually in 3–7 districts and 6–35 municipalities. In peak years (2013, 2018, 2022), all districts reported cases, with 21–35 municipalities affected.

**Figure 3:**
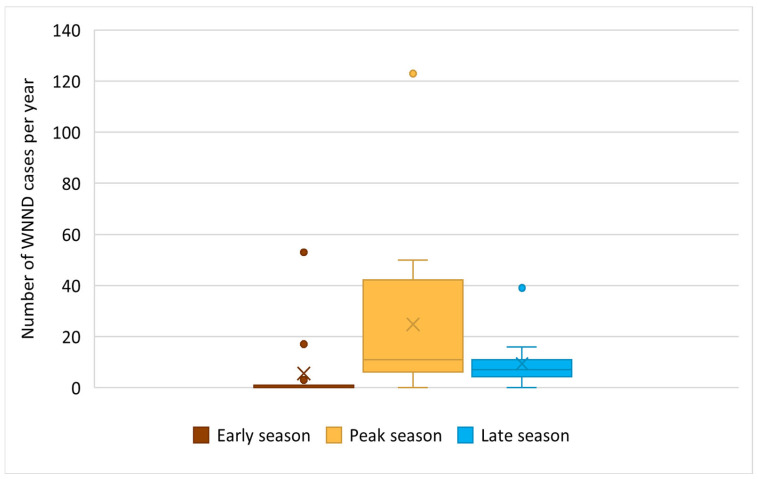
Seasonal distribution of cumulative WNND cases per year across early (epidemiological weeks 24–27), peak (weeks 28–35), and late (weeks 36–40) transmission periods in Vojvodina, Serbia, 2012–2025. Boxplots show median and interquartile range; × denotes the sample mean and • denotes outliers.

Seasonal stratification into early, peak, and late transmission periods revealed differences in the cumulative annual burden of WNND, with the highest number of cases occurring during the peak transmission period (Figure 3). Week-specific analysis further characterized this pattern, demonstrating a marked concentration of cases during epidemiological weeks 31–34 (Figure S2).

All patients were hospitalized. No cases were associated with blood donation, transfusion or organ transplantation, and none of the patients had been vaccinated against *Ortoflavivirus* infections. Clinical presentations of WNND varied over the study period, with encephalitis as the predominant manifestation (64.63%) (Table 3). Meningoencephalitis and meningitis accounted for most remaining cases, while a small proportion (<3%) were classified as unspecified WNND. Notably, no cases of AFP associated with WNV infection were reported, including among these unspecified cases. Annual CFRs ranged from 0.00% to 25.00%, with a median of 11.56% (IQR:7.69–16.67). The highest median annual CFRs were observed among encephalitis (12.15%) and meningoencephalitis cases (10.00%), whereas meningitis and unspecified WNND had median annual CFRs of 0.00%, reflecting the predominance of years without fatal outcomes.

Spearman correlation analysis showed a strong positive association between annual meningoencephalitis cases and annual CFR (ρ = 0.66, *p* = 0.01), and a moderate positive association for unspecified WNND cases and annual CFR (ρ = 0.56, *p* = 0.04) (Table S2). These results indicate that years with higher numbers of these clinical presentations were associated with increased lethality. During the COVID-19 pandemic period (2021–2023), a total of 139 WNND cases were registered. SARS-CoV-2 infection was identified in 9.68% of WNND survivors (12/124) and in 13.33% of fatal cases (2/15), either as a co-infection or occurring within one month before WNND onset or during the one-month recovery period.

Non-survivors (n = 72) were older and had more comorbidities than survivors (n = 485). In univariable analysis, age, comorbidity burden (including higher CCI), and the presence of cardiovascular disease, diabetes, malignancy, and leukemia were significantly associated with WNND-related mortality (Table 4).

In multivariable logistic regression analysis, a higher CCI was independently associated with a fatal outcome in WNND patients (OR 1.52, 95% CI 1.27–1.82; *p* < 0.001). In addition, cardiovascular disease (OR 2.10, 95% CI 1.08–4.07; *p* = 0.029), diabetes mellitus (OR 2.32, 95% CI 1.26–4.18; *p* = 0.005), and malignancy (OR 4.21, 95% CI 1.62–10.90; *p* = 0.003) remained significant independent predictors of fatal outcome (Table 5). Leukemia, although significant in the univariable analysis, did not retain statistical significance in the multivariable model (Table 5).

## 4. Discussion

This retrospective, population-based study provides a 14-year overview of WNND in Vojvodina. The high proportion of laboratory-confirmed cases, along with the predominance of autochthonous infections, underscore the ongoing endemic WNV transmission in Vojvodina. In addition, the detection of WNV-specific IgM in both serum and CSF among confirmed cases highlights the important role of CSF antibody testing for definitive diagnosis. The relatively low detection rate of WNV RNA, particularly in serum, reflects the short viremic phase and possible late presentation of WNND. Approximately half of confirmed cases had serum IgG antibodies, indicating a more advanced immune response at sampling. In probable cases, the presence of WNV-specific IgM and IgG antibodies in serum, combined with the absence of intrathecal IgM or WNV RNA in CSF, likely reflects weak intrathecal immunity, early or limited central nervous system involvement, or suboptimal CSF sampling. These findings emphasize the diagnostic value of CSF-based testing in WNND confirmation.

The pronounced interannual variability in WNND incidence and mortality in Vojvodina reflects an endemic–epidemic pattern. Peaks in WNND incidence, recorded in 2013, 2018 and 2022 indicate intensive WNV transmission and a higher burden of disease in the human population. The absence of reported cases in 2020 likely reflects underreporting due to disruptions in surveillance, healthcare access, and diagnostic capacity during the first year of the COVID-19 pandemic, rather than a true absence of cases, as observed across much of Europe [10,35].

Within Southeastern Europe, Serbia is among the non-EU countries with the highest reported burden of WNF, frequently reporting high numbers of human cases, particularly in epidemic years [19,20,21,36]. Although the national enhanced surveillance was designed to monitor WNF broadly [37], operative reporting in practice has focused primarily on WNND, as these cases are more consistently recognized, hospitalized, and laboratory-confirmed. Between 2012 and 2023, a total of 1388 WNF cases were reported in Serbia, with annual counts ranging from 30 cases in 2015 to a peak of 415 cases in 2018; notably, no cases were reported in 2020 [38,39]. During 2012–2025, reported WNF incidence in neighboring countries was generally lower than in Serbia, with rates of approximately 0.2–2.3 per 100,000 in Hungary, 0.03–1.4 per 100,000 in Romania, and 0.00–1.52 per 100,000 in Croatia, whereas Serbia reported higher rates, reaching up to 5.80 per 100,000 [21]. The highest incidence in all four countries occurred in 2018 [19]. Differences in WNF incidence between southeastern countries may reflect migratory bird movements but are also influenced by microclimatic conditions, surveillance practices, and underreporting [6,21,36]. During the specified period, over one third of all reported cases in Serbia (n = 505; 36.38%) occurred in Vojvodina [39]. High incidence rates in Vojvodina and the City of Belgrade are supported by data from Serbia’s national integrated WNV surveillance program (launched in 2014), which documented the highest virus circulation in these areas using sentinel animals, mosquitoes, and wild birds [40]. These findings underscore the link between WNV enzootic activity and increased human infection, further supported by evidence of intensive WNV circulation in horses and mosquitoes in Vojvodina prior to the implementation of integrated surveillance [20,40,41]. It should be noted that molecular characterization studies conducted in Vojvodina identified WNV lineage 2 as the strain responsible for human WNF cases in the period 2012–2015 [42]. In addition, integrated surveillance in Vojvodina showed continuous circulation of WNV lineage 2 in multiple hosts, mosquitoes, birds, horses and humans, supporting the presence of an established enzootic WNV transmission cycle in this area [43].

The 2018 WNV season was characterized by an unusually early onset and intensified transmission across Southeast Europe, including Serbia, Greece, Romania, Hungary, and Croatia, reflecting a synchronized regional increase in endemicity likely driven by shared climatic and ecological factors [19,20]. Consistent with this pattern, Vojvodina—reflecting national trends—recorded the highest number of cases in 2018, along with the longest transmission period and the widest spatial distribution of cases since the introduction of WNF surveillance in 2012 [19,20,38]. The peak incidence and mortality were likely influences by exceptionally high early-spring temperatures, with April 2018 being the warmest in Serbia since 1888 [20]. These conditions likely promoted early mosquito activity, an extended transmission season, and intensified virus amplification among vectors and hosts. Integrated surveillance detected WNV in 12.65% of mosquito samples (exceeding 20% in six districts) and in wild birds (10.13% in dead birds, 6.56% in live birds) [20,40]. Notably, WNV detection in mosquitoes, horses, and birds preceded human cases by up to eight weeks, highlighting their predictive value for early warning.

The WNND-related mortality observed in Vojvodina, with a mean rate of 0.28 per 100,000 population and a mean CFR of 12.93%, falls within the range reported in other parts of Southeastern Europe, such as Greece and Italy [44,45]. Although the overall CFR during a recent large WNF outbreak in Italy was approximately 9% [45,46], substantially higher fatality rates—up to 17.9%—have been documented among patients with WNND in Europe [47]. In the multinational study spanning 10 countries, including Serbia and three neighboring countries, Popescu et al. reported a mean WNND-associated CFR of 16.36% during 2010–2017 [48].

In our dataset, males exhibited higher incidence and mortality rates than females, consistent with numerous studies showing that severe WNND predominantly affects older males [10,11,49]. This male predominance may reflect greater exposure to outdoor activities during peak mosquito activity and potential sex-based differences in immune response. The median age of patients was 65 years (IQR: 55–72), comparable to other multinational and regional studies, which report median ages ranging from 60 years (IQR: 43.5–72.0) [48] to 76 years (range: 14–96) [44]. Likewise, Nikolić et al. found that 67.7% of WNND patients hospitalized during the 2022 season at the University Clinical Center of Serbia were older than 65 years [28], while 63.91% of our cohort were older than 60. The marked increase in incidence and mortality with age—particularly among those over 70— reinforces established risk gradients in WNND, where older age is consistently associated with higher rates of neuroinvasive disease and death. Comparable age-associated mortality patterns have been reported in Mediterranean countries, highlighting the disproportionate burden of WNND among the elderly and those with comorbidities [44,48,49].

In our study, marked spatial heterogeneity in average WNND incidence and mortality was observed at the district level. The higher WNND incidence in South Banat and to a lesser extent South Bačka, likely reflects favorable ecological and climatic conditions, where the overlap of *Culex pipiens* mosquitoes and abundant bird populations enables sustained WNV enzootic amplification and increased spillover risk to humans [43,50]. South Banat contains extensive networks of rivers, standing waters, irrigation canals, and floodplains, providing ideal mosquito breeding habitats. Spatial analyses further identified hydrographic factors—such as the presence of rivers and streams, proximity to water bodies, and wild bird habitats— as strong predictors of WNV clusters in the district [50]. In addition, South Banat lies along key bird migration routes and provides suitable habitats for both resident and migratory birds, which act as primary virus reservoirs [51].

Areas with frequent bird activity facilitate the introduction and maintenance of WNV within the mosquito-bird cycle, increasing the risk of spillover to humans. National surveillance has documented intensive WNV circulation in areas with such ecological conditions [20,39,40,50]. Vojvodina’s flat lowland terrain, hot summer continental climate, and extensive network of slow-flowing rivers, wetlands, and lakes provide optimal habitats for large populations of *Culex pipiens* mosquitoes and resident wild birds. These ecological features, combined with the role of migratory birds in virus introduction, make Vojvodina a persistent WNV hotspot compared with other Balkan regions [20,39,40].

The high proportion of recent, concurrent, or subsequent SARS-CoV-2 infection among WNND patients during the COVID-19 pandemic may reflect older age and a higher burden of comorbidities, which are common risk factors for severe outcomes in both COVID-19 and WNND [52,53]. Moreover, immune dysregulation associated with acute or recent SARS-CoV-2 infection, including lymphopenia and impaired antiviral responses, could have increased susceptibility to severe WNND or delayed recovery [54,55]. Finally, the strain on healthcare systems during the pandemic, including delayed hospital presentation, diagnostic problems, and limited access to intensive care, likely contributed to poorer outcomes in this subgroup of WNND patients [56].

Encephalitis and meningoencephalitis were the most common clinical forms of WNND in Vojvodina, consistent with other studies [28,57]. Although fatalities occurred predominantly among patients with these two entities, severe outcomes, while rare, were observed across all clinical WNND forms [57,58]. The interannual variation in WNND mortality probably reflects not only disease severity but also differences in outbreak intensity, patient age distribution, and comorbidity prevalence [57,58,59]. Positive correlations indicate that years with higher proportions of severe or poorly defined WNND presentations, such as meningoencephalitis and unspecified forms, were associated with increased overall CFRs, reflecting the influence of disease severity and diagnostic uncertainty rather than a direct causal relationship. Given the low number of fatal cases among unspecified WNND, this correlation should be considered primarily as a statistical observation rather than evidence of a meaningful clinical association. Meningoencephalitis is inherently linked to a worse prognosis [57,60], while unspecified WNND cases may reflect late diagnosis, limited clinical data, or strained healthcare systems during peak WNV transmission [61]. These patterns should be interpreted alongside patient age, comorbidities, healthcare availability, and epidemic intensity [49,62].

Combined seasonal and week-specific analyses revealed a pronounced and consistent temporal pattern of WNND in Vojvodina. The highest cumulative annual burden occurred during the peak transmission period, with week-specific analysis localizing this peak to epidemiological weeks 31–34 (early–mid August). Similar seasonal clustering has been reported in other European countries, including Italy [17], Greece [44], Hungary [63], and Croatia [64], where peak WNF incidence typically occurs between late July and early September. The aligned timing regionally suggests common ecological and environmental determinants of WNV transmission [19]. High summer temperatures, periods of reduced precipitation followed by episodic rainfall increase mosquito abundance, accelerate viral replication within vectors, and increase human exposure [65,66,67]. The mid-season clustering of cases therefore likely reflects optimal climatic conditions for WNV amplification in mosquito populations, as well as increased vector–host contact during the warmest months [67]. These findings underscore the importance of intensifying surveillance, vector control, and public health preparedness during this relatively narrow window of heightened WNV transmission risk.

Numerous studies have shown that, in addition to older age, chronic underlying diseases and immunosuppression substantially increase the risk of severe outcomes and mortality following WNV infection [10,11,49,62]. In our study, non-survivors were significantly older and had a higher comorbidity burden than survivors. Univariable analysis identified age, number of comorbidities, higher CCI, cardiovascular disease, diabetes mellitus, malignancy, and leukemia as factors associated with mortality. However, multivariable logistic regression showed that only higher CCI, cardiovascular disease, diabetes mellitus, and malignancy remained independent predictors of fatal outcome. A one-point higher CCI conferred 1.52-fold higher odds of death, while cardiovascular disease, diabetes mellitus, and malignancy were associated with 2.10-fold, 2.32-fold, and 4.21-fold higher odds of death, respectively. Leukemia lost significance after adjustment, suggesting its effect is largely mediated by overall comorbidity burden. Notably, age and sex were not independent predictors, indicating that comorbidity burden rather than chronological age is a key determinant of mortality in WNND. These findings align with previous studies identifying comorbidity, age, and neurological severity as predictors of adverse outcomes [48,53,58].

Overall WNND mortality is approximately 10% but may reach 20% among individuals aged ≥70 years and 30–40% in patients with solid organ transplantation, hematological malignancies, or those receiving B-cell–depleting therapies [59]. These findings underscore the importance of early recognition and careful management of patients with multiple comorbidities, particularly cardiovascular disease, diabetes, and malignancy [11,68]. Public health strategies should emphasize case detection, risk communication, personal protective measures and community-based mosquito control to reduce exposure and disease severity.

This study has a number of limitations. Its retrospective design and reliance on surveillance data may have resulted in incomplete clinical information and underreporting, particularly of less severe WNND cases. The analysis was restricted to WNND and therefore does not capture the full clinical spectrum of WNV infections in the population, including WNF and AFP. For the purpose of identifying predictors of mortality, outcomes were dichotomized as survivors versus non-survivors. Although some survivors experienced persistent neurological sequelae, investigation of determinants of persistent disability is outside the scope of the present study and warrants future research.

The lack of seroprevalence data makes it impossible to estimate the proportion of asymptomatic WNV infections in the general population. The low rate of WNV PCR positivity likely reflects the short duration of viremia and variability in the timing of sample collection. Furthermore, delayed clinical recognition and late submission of samples may have further decreased PCR detection in serum and CSF. According to the ECDC case definition [28], serological results must be interpreted in the context of prior *Ortoflavivirus* exposure and vaccination history; confirmation in such cases requires virus neutralization testing (e.g., plaque reduction neutralization test) or an equivalent assay. Currently, the national reference laboratory for arboviruses does not perform neutralization testing [39], which may limit specificity because serological cross-reactivity with co-circulating *Ortoflaviviruses* (e.g., Usutu virus) cannot be fully excluded. Nevertheless, the WNND surveillance data presented here—based on neurologically manifesting cases and national/EU case definitions—together with contemporaneous integrated surveillance findings from mosquitoes, wild birds and sentinel animals [20,40], support sustained endemic circulation of WNV in Vojvodina. The long study period encompassed changes in surveillance practices, diagnostic capacity, and healthcare system organization, particularly during the COVID-19 pandemic, which may have influenced case detection and reporting. Although temporal trends were examined, none reached statistical significance, likely due to limited annual case numbers and marked interannual variability. Associations between clinical forms of WNND and CFRs were based on aggregated population-level data and should therefore be interpreted as ecological rather than individual-level associations. Finally, comorbidity data were extracted from medical records and may be subject to residual confounding despite multivariable adjustment.

Nevertheless, this population-based study provides a robust overview of WNND in Vojvodina over a 14-year period, supported by a large number of laboratory-confirmed cases. The comprehensive epidemiological and clinical dataset enabled reliable assessment of temporal, seasonal, and spatial patterns, as well as identification of independent predictors of fatal outcome, thereby strengthening the relevance of these findings for WNF surveillance and public health planning in Serbia.

## 5. Conclusions

The results of the study demonstrate that WNND remains a significant public health concern in Vojvodina, Serbia, characterized by pronounced interannual variability, seasonal clustering during periods of peak mosquito activity, and substantial mortality. The disease predominantly affected older adults, especially males, with both incidence and fatal outcomes increasing with age. Importantly, a higher comorbidity burden, particularly cardiovascular disease, diabetes mellitus, and malignancy, was independently associated with fatal outcome, underscoring the need for early recognition and careful clinical management of high-risk patients. The observed geographic heterogeneity and recurring epidemic peaks highlight WNND as an important indicator for surveillance and for guiding timely public health interventions. Reinforcing laboratory capacity for virus neutralization testing and expanding targeted serological surveys at the national level would contribute to the specificity of case confirmation and improve future surveillance. Strengthening integrated surveillance, targeted prevention, and risk communication especially for vulnerable high-risk populations, remain essential to reduce the impact of WNV infection in the study area.

## Figures and Tables

**Table 1:** Laboratory diagnostic results among confirmed and probable WNND cases in Vojvodina, Serbia, 2012–2025 ^1^.

Diagnostic Marker	Type of Sample	Number of Tested	Laboratory-Confirmed WNND (*n* = 530)	Probable WNND (*n* = 27)
WNV RNA		N	Positive *n* (% of tested)	Positive *n* (% of tested)
	Serum	101	20 (19.80)	0 (0)
	CSF	422	62 (14.69)	0 (0)
Serology	IgM Serum	1337	530 (39.64)	27 (2.02)
	IgG Serum	1337	229 (17.12)	27 (2.02)
	IgM CSF	1337	530 (39.64)	0 (0)

^1^ All suspected cases (*n* = 1337) had paired serum and CSF samples tested.

**Table 2:** WNND incidence and mortality by age, sex and district, Vojvodina, Serbia 2012–2025.

Characteristic	Total Cases *n* (%)	Mean Incidence Rate ^1^ (95% CI)	Mean Mortality Rate ^1^ (95% CI)
Sex			
Male	351 (63.02)	2.81 (0.81–4.82)	0.40 (0.002–0.79)
Female	206 (36.98)	1.57 (0.38–2.75)	0.17 (0.03–0.31)
District of Vojvodina			
South Bačka	180 (32.32)	2.09 (0.15–4.03)	0.34 (0.00–0.75) ^2^
Srem	78 (14.00)	1.89 (0.68–3.10)	0.26 (0.00–0.52)
North Bačka	14 (2.51)	0.57 (0.21–0.93)	0.00 (NA) ^3^
West Bačka	33 (5.92)	1.45 (0.32–2.58)	0.09 (0.00–0.22) ^2^
North Banat	21 (3.77)	1.17 (0.15–2.18)	0.17 (0.00–0.36) ^2^
Central Banat	37 (6.64)	1.59 (0.50–2.69)	0.13 (0.00–0.34) ^2^
South Banat	194 (34.84)	4.97 (1.16–8.77)	0.61 (0.04–1.18)
Age group (years)			
0–9	0 (0.00)	0.00 (NA)	0.00 (NA)
10–19	9 (1.61)	0.34 (0.00–0.70) ^2^	0.00 (NA)
20–29	16 (2.87)	0.51 (0.00–1.06) ^2^	0.00 (NA)
30–39	30 (5.39)	0.85 (0.23–1.48)	0.00 (NA)
40–49	44 (7.90)	1.23 (0.15–2.32)	0.06 (0.00–0.14) ^2^
50–59	102 (18.31)	2.80 (0.95–4.66)	0.26 (0.00–0.53) ^2^
60–69	168 (30.16)	4.63 (1.09–8.17)	0.42 (0.11–0.73)
70–79	137 (24.60)	5.97 (1.50–10.45)	1.19 (0.00–2.41) ^2^
>80	51 (9.16)	5.09 (1.60–8.59)	1.78 (0.00–3.80) ^2^
Total	557 (100.00)	2.17 (0.60–3.75)	0.28 (0.02–0.53)

^1^ Per 100,000 population; ^2^ Lower confidence limits were truncated at zero where negative values occurred; ^3^ NA-indicates that the 95% confidence interval was not calculated due to zero events.

**Table 3:** Clinical forms of WNND and corresponding CFRs in Vojvodina, Serbia, 2012-2025.

Clinical Form of WNND	Cases *n* (%)	Mean Annual Proportion of Cases ^1^ % (95% CI) ^2^	Median Annual CFR ^3^, % (IQR: Q1–Q3) ^4^
Encephalitis	360 (64.63)	59.50 (46.10–72.90)	12.15 (0.00–16.67)
Meningoencephalitis	105 (18.85)	18.70 (11.20–26.10)	10.00 (0.00–33.33)
Meningitis	76 (13.64)	13.30 (8.10–18.50)	0.00 (0.00–0.00)
Unspecified WNND	16 (2.88)	1.40 (0.00–3.00)	0.00 (0.00–0.00)
Total	557 (100)	NA ^5^	11.56 (7.69–16.67)

^1^ Mean annual proportion of cases calculated relative to the total annual number of WNND cases. ^2^ 95% confidence interval. Lower limits were truncated at zero where negative values occurred. ^3^ Case fatality rate. ^4^ Interquartile range (Q1–Q3). ^5^ NA—not applicable; total annual proportion equals 100% by definition and was not summarized with a mean or confidence interval.

**Table 4:** Predictors of fatal outcome in WNND, Vojvodina, Serbia, 2012–2025.

Variable	Survivors (n = 485)	Non-Survivors (n = 72)	Univariable OR ^3^ (95% CI) ^4^	*p* Value
	*n* (%) or Mean ^1^ (95% CI) ^2^	*n* (%) or Mean (95% CI)		
Age (years)	60.54 (59.19–61.90)	70.83 (68.44–73.23)	1.07 (1.04–1.09)	<0.001
Sex (male)	301 (62.10)	50 (69.40)	1.39 (0.81–2.37)	0.227
Number of comorbidities	1.22 (1.13–1.31)	1.99 (1.79–2.21)	1.93 (1.52–2.45)	<0.001
Charlson Comorbidity Index (CCI)	3.09 (2.91–3.27)	5.07 (4.72–5.41)	1.78 (1.52–2.09)	<0.001
Cardiovascular disease ^5^	64 (13.20)	23 (31.90)	3.09 (1.76–5.41)	<0.001
Diabetes mellitus	100 (20.60)	36 (50.00)	3.85 (2.31–6.42)	<0.001
Malignancy ^6^	15 (3.10)	11 (15.30)	5.64 (2.48–12.83)	<0.001
Hypertension	260 (53.60)	44 (61.10)	1.36 (0.82–2.26)	0.234
Chronic obstructive pulmonary disease	53 (10.90)	10 (13.90)	1.32 (0.64–2.72)	0.460
Chronic kidney disease	25 (5.20)	1 (1.40)	0.26 (0.04–1.94)	0.189
Chronic rheumatic disease	18 (3.70)	5 (6.90)	1.94 (0.70–5.39)	0.206
Cerebrovascular insult	21 (4.30)	2 (2.80)	0.63 (0.15–2.75)	0.540
Myocardial infarction	27 (5.60)	7 (9.70)	1.87 (0.77–4.36)	0.175
Leukemia	8 (1.70)	4 (5.60)	3.49 (1.02–11.91)	0.046

^1^ Data are presented as mean (95% CI) deviation or number (percentage), as appropriate. Continuous variables expressed as mean (95% CI), categorical as *n* (%); ^2^ CI, confidence interval; ^3^ OR, odds ratio; ^4^ variables statistically significant in univariable analysis are shown in bold; ^5^ excluding myocardial infarction; ^6^ excluding leukemia.

**Table 5:** Independent predictors of fatal outcome in WNND patients in Vojvodina, Serbia, 2012–2025.

Variable	Survivors (n = 485) *n* (%) or Mean ^1^ (95% CI) ^2^	Non-Survivors (n = 72) *n* (%) or Mean (95% CI)	Multivariable ^3^ OR ^4^ (95% CI)	*p*-Value
Charlson Comorbidity Index (CCI)	3.09 (2.91–3.27)	5.07 (4.72–5.41)	1.52 (1.27–1.82)	<0.001
Cardiovascular disease ^5^	64 (13.20)	23 (31.90)	2.10 (1.08–4.07)	0.029
Diabetes mellitus	100 (20.60)	36 (50.00)	2.32 (1.26–4.18)	0.005
Malignancy ^6^	15 (3.10)	11 (15.30)	4.21 (1.62–10.9)	0.003

^1^ Data are presented as mean (95% CI) deviation or number (percentage), as appropriate. Continuous variables expressed as mean (95% CI), categorical as n (%); ^2^ CI, confidence interval; ^3^ multivariable logistic regression analysis; ^4^ OR, odds ratio; ^5^ excluding myocardial infarction; ^6^ excluding leukemia.

## Data Availability

The data underlying the results of this study can be obtained from the corresponding author upon reasonable request.

## Supplementary Materials

The following supporting information can be downloaded at: https://www.mdpi.com/article/10.3390/v18030312/s1, Table S1. Average annual percent change (AAPC) in the incidence and mortality rate (per 100,000 population) of WNND in the period 2012–2025 in Vojvodina, Serbia; Table S2. Correlation of annual number of different clinical forms of WNND and annual case-fatality rate (CFR), in Vojvodina, Serbia (2012–2025); Figure S1. (A) Joinpoint regression analysis plot of WNND incidence rate per 100.000 population trend in the period 2012–2025 in Vojvodina, Serbia. (B) Joinpoint regression analysis plot of WNND mortality rate trend in the period 2012–2025 in Vojvodina, Serbia; Figure S2. Seasonal distribution of cumulative WNND cases by epidemiological weeks 24-40 in Vojvodina, Serbia (2012–2024). Box-and-whisker plots summarize the distribution of cumulative WNND cases across seasons for each epi week. Boxes represent the interquartile range (IQR), the central line indicates the median, and whiskers denote the minimum and maximum values.
