# Supplementary material for: Spatiotemporal and Demographic Patterns of West Nile Neuroinvasive Disease in Vojvodina, Serbia, 2012–2025
Source: Viruses. 2026 Mar 2;18(3):312. doi: 10.3390/v18030312 (PMC13030431; doi:10.3390/v18030312)
Supplement: Supplementary file 1 [file viruses-18-00312-s001.zip › viruses-4150906-supplementary.pdf]

Supplement

Table S1. Average annual percent change (AAPC) in the incidence and mortality rate (per 100,000 population) of WNND in the period 2012–2025 in Vojvodina, Serbia.

Rate/100,000	AAPC	Lower 95%CI[1]	Upper 95%CI[1]	p value
**Incidence**	3.89	-25.21	44.32	0.82
**Mortality**	-12.1	-85.88	447.16	0.89

[1] The AAPC confidence interval is based on the normal distribution.

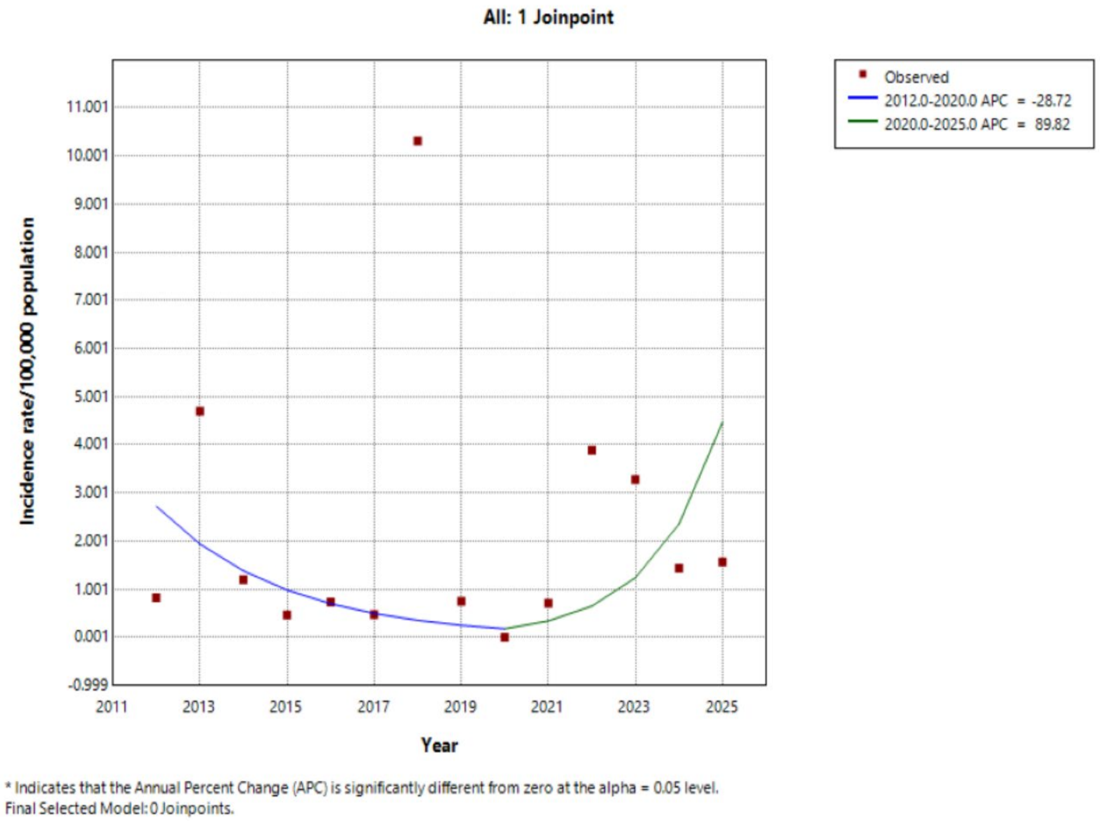

(A)

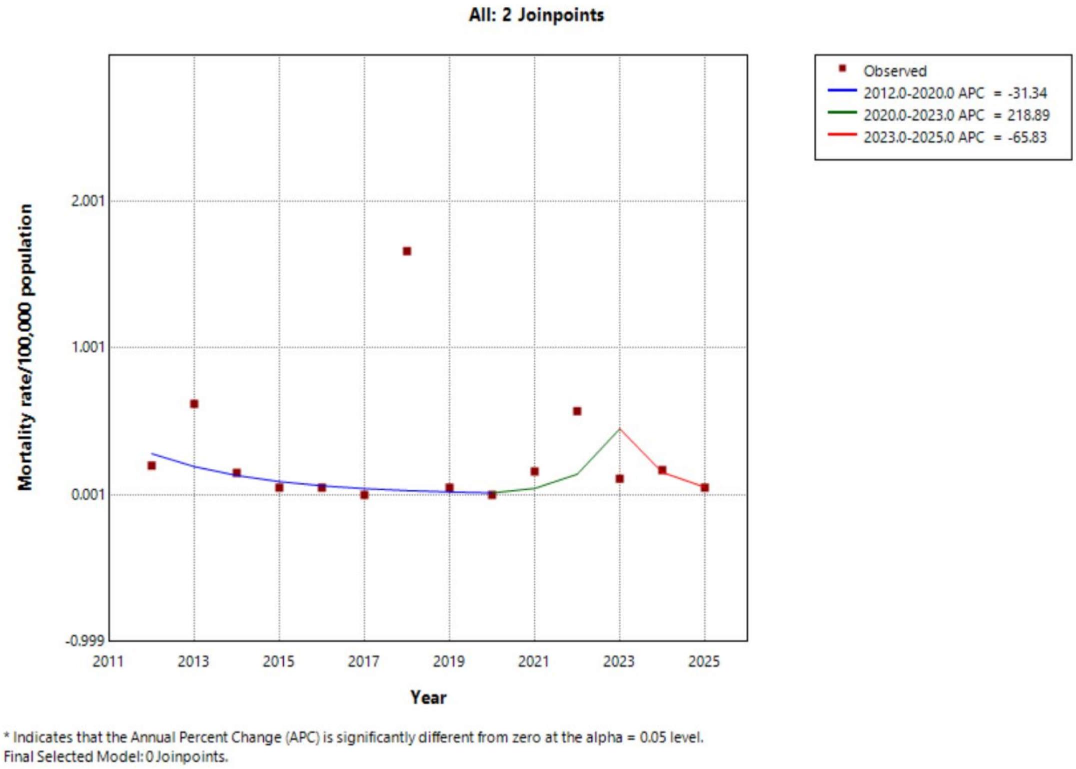

(B)

Figure S1. (**A**) Joinpoint regression analysis plot of WNND incidence rate per 100.000 population trend in the period 2012-2025 in Vojvodina, Serbia. (**B**) Joinpoint regression analysis plot of WNND mortality rate trend in the period 2012-2025 in Vojvodina, Serbia.

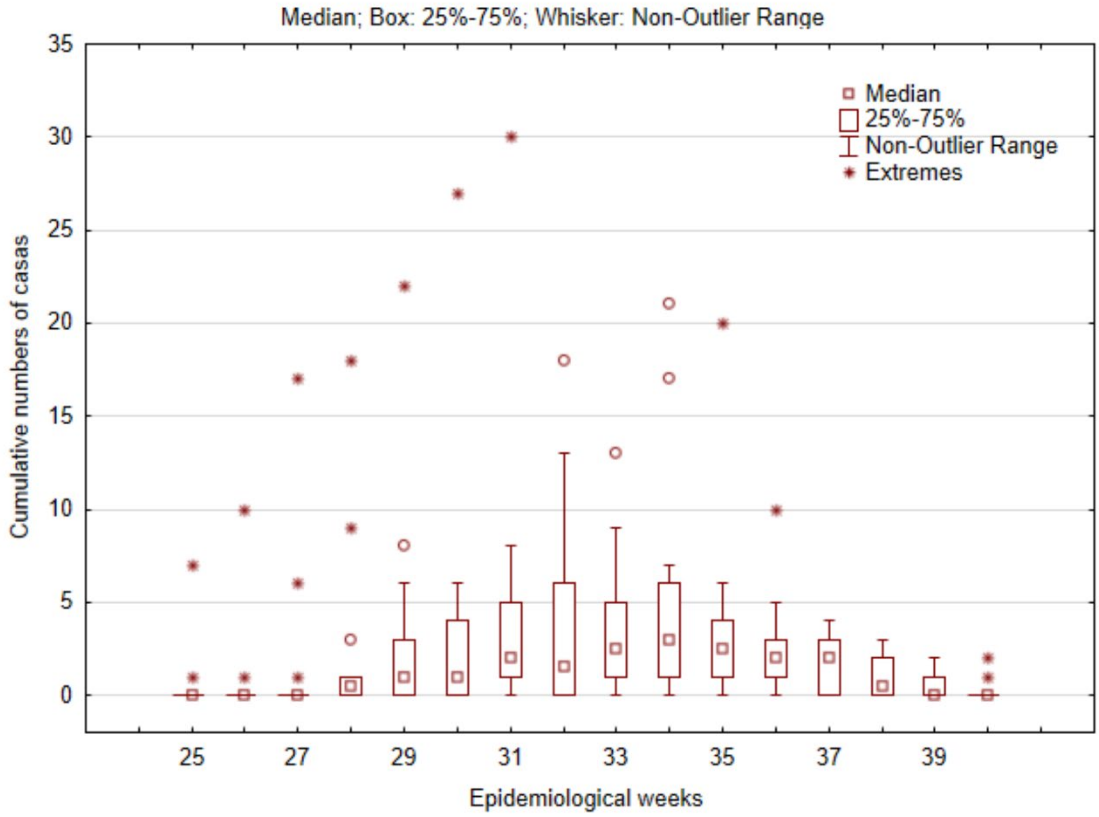

Figure S2. Seasonal distribution of cumulative WNND cases by epidemiological weeks 24-40 in Vojvodina, Serbia (2012–2024). Box-and-whisker plots summarize the distribution of cumulative WNND cases across seasons for each epi week. Boxes represent the interquartile range (IQR), the central line indicates the median, and whiskers denote the minimum and maximum values.

Table S2. Correlation of annual number of different clinical forms of WNND and annual case-fatality rate (CFR), in Vojvodina, Serbia (2012–2025).

Clinical form of WNND	WNND cases (n=557)	Spearman ϱ	p value
**CFR (%)**	/	1.00	/
**Encephalitis**	360	0.399	0.158
**Meningoencephalitis**	105	0.657	**0.011**
**Meningitis**	76	0.287	0.319
**Unspecified WNND**	16	0.556	**0.039**
